# Serum steroid and thyroid hormone concentrations in healthy domestic male and female alpacas

**DOI:** 10.3389/fvets.2023.1281053

**Published:** 2023-12-08

**Authors:** Kellie Fecteau, Luca Giori, Hugo Eiler, Alex Esteller-Vico, Andrea Lear, Ricardo Videla

**Affiliations:** ^1^Department of Biomedical and Diagnostic Sciences, College of Veterinary Medicine, University of Tennessee, Knoxville, TN, United States; ^2^Department of Large Animal Clinical Sciences, College of Veterinary Medicine, University of Tennessee, Knoxville, TN, United States

**Keywords:** alpaca, concentration, hormone, steroid, thyroid

## Abstract

Alpacas are high quality fiber producing animals, kept for production purpose and as pets. Endocrine imbalances from adrenal glands, gonads, or thyroid gland may result in coat abnormalities in domestic animals and affect reproduction. Contrary to many domesticated animals, information on hormone concentrations in alpacas is scarce. The purpose of this study was to provide steroid and thyroid hormone values in domestic alpacas. Blood was collected from healthy male (35 intact, 2 castrated) and female (48 non-pregnant, 3 pregnant) alpacas from local farms in Tennessee. Adrenal, reproductive, and thyroid hormones were analyzed. There were no significant differences in median concentrations of progesterone, estradiol, thyroxine (T4), and triiodothyronine (T3) between intact male and female non-pregnant alpacas. Median concentrations of testosterone, androstenedione, 17-hydroxyprogesterone, and cortisol were significantly higher in intact male alpacas compared to female non-pregnant alpacas. This information provides adrenal, gonadal, and thyroid hormone concentrations in alpacas to help with diagnosis of endocrine disorders.

## Introduction

Alpacas (*Lama pacos*) belong to the *Camelidae* family and are high quality fiber producing animals native to South America. Alpacas have gained popularity in countries outside of South America including Great Britain, the United States, Australia (1–3), and Canada (1, 4). Alpacas were imported from South America to the United States in 1984 (4), mainly for fiber production and breeding (5). As of 2017, there were 121,904 alpacas on 10,054 farms in the United States (6). According with the 2017 Census of Agriculture, the number of farms with alpacas had increased since the previous census in 2012 however the number of alpacas decreased, which may be suggestive of a shift in the alpaca’s value from a production animal to a companion animal. The increased popularity of alpacas around the world, either as pets or production animals, increases the need for veterinary care. Contrary to many domesticated species, information on hormone concentrations other than progesterone and estradiol (7–9) in alpacas is scarce. Progesterone analysis is integral to any breeding program however, analyses of other hormones, such as cortisol and thyroid hormones are of value when evaluating reproductive health and general health of the animal (10, 11). Endocrine imbalances from adrenal glands, gonads, or thyroid gland may result in coat abnormalities, affect reproduction, and affect the general health and/or behavior of domestic animals (12–15). The purpose of this study was to determine the concentrations of adrenal and gonadal steroids and thyroid hormones in healthy domestic male and female alpacas, which may contribute to the development of reproductive and other endocrine diagnostic tests, such as the adrenal panel our laboratory offers for other domestic species (16–19), and increase our knowledge of alpaca physiology.

## Materials and methods

### Animals

Eighty-eight healthy Huacaya and Suri alpacas (35 intact and 2 castrated males, age 1–15 years; 51 intact females, age 2–12 years) from privately owned farms in Tennessee were used in this study. Five of the 35 intact male alpacas were less than 2 years of age and considered prepubertal. The study was conducted from August to October 2015. A physical examination was performed on each alpaca, which included a body condition and FAMACHA score, fecal exam, body temperature, and complete blood count and biochemical panel. Alpacas were removed from the study if mucous membranes were pale, body condition score was poor (<3 out of 5), or if they had fever. All farms were visited between 8 AM and 12 PM. Alpacas were not fasted prior to blood collection and had diets comprised of pellets, hay, and minerals (free access). Alpacas were housed either in outdoor pens with a shelter or in pasture with a barn. Animals were restrained by an assistant or in a chute on the farm while blood was collected from the jugular vein and placed in a red top vacuum tube. The study protocol was approved by the University of Tennessee’s Institutional Animal Care and Use Committee (protocol 2298–0914). Blood samples were kept in a cooler with ice (no more than 5 h) until return to the laboratory where they were centrifuged and serum frozen at −80°C. Hormones were analyzed within 2 months of collection.

### Hormone analysis

All samples were analyzed together in the same assay for each hormone in order to decrease variability. Analysis of cortisol, progesterone, testosterone, T4, and T3 was performed using chemiluminescent methodology (Immulite 1000, Siemens Medical Solutions USA, Inc. Malvern, PA). Analysis of estradiol, androstenedione, and 17-hydroxyprogesterone was performed using radioimmunoassay methodology (ImmunChem Double Antibody, MP Biomedicals, Costa Mesa, CA, United States). Hormones analyzed using radioimmunoassay were analyzed in duplicate and hormones analyzed using chemiluminescent methodology were measured by the instrument 10 times. For evaluation of kit performance in alpaca serum, intra-assay and inter-assay coefficients of variation (CV) and spike recovery were determined in pooled alpaca serum. Spike recovery was performed by adding either assay control material (Lyphochek Immunoassay Plus Control, Biorad) for Immulite assays or assay standard material for radioimmunoassays. Intra- and inter-assay CVs for cortisol were 7.8% and 10.5%, respectively. Spiking recovery with known amount of control material added to alpaca serum was 104.4%. Analytical sensitivity of the assay was 0.2 μg/dL based on information provided by the manufacturer. Intra- and inter-assay CVs for progesterone were 6.50% and 11.9%, respectively. Spiking recovery with known amount of control material added to alpaca serum was 102.1%. Analytical sensitivity of the assay was 0.2 ng/mL based on information provided by the manufacturer. Intra- and inter-assay CVs for testosterone were 7.9% and 10.6%, respectively. Spiking recovery with known amount of control material added to alpaca serum was 80.9%. Analytical sensitivity of the assay was 15.0 ng/dL based on information provided by the manufacturer. Intra- and inter-assay CVs for estradiol were 22.1% and 27.6%, respectively. Spiking recovery with known amount of estradiol standard added to alpaca serum was 78.9%. Analytical sensitivity of the assay was 7.2 pg./mL based on information provided by the manufacturer. Intra- and inter-assay CVs for androstenedione were 13.9% and 14.6%, respectively. Spiking recovery with known amount of androstenedione standard added to alpaca serum was 71.8%. Analytical sensitivity of the assay was 0.05 ng/mL based on information provided by the manufacturer. Intra- and inter-assay CVs for 17-hydroxyprogesterone were 6.4 and 10.0%, respectively. Spiking recovery with known amount of 17-hydroxyprogesterone standard added to alpaca serum was 101.0%. Analytical sensitivity of the assay was 0.08 ng/mL based on information provided by the manufacturer. Intra- and inter-assay CVs for T4 were 3.7% and 7.3%, respectively. Spiking recovery with known amount of control material added to alpaca serum was 95.7%. Analytical sensitivity of the assay was 0.12 μg/dL based on information provided by the manufacturer. Intra-assay and inter-assay CVs for T3 were 7.4% and 10.6%, respectively. Spiking recovery with known amount of control material added to alpaca serum was 103.1%. Analytical sensitivity of the assay was 35 ng/dL based on information provided by the manufacturer.

### Statistical analysis

Statistical analyses were performed with a commercially available software package (JMP Pro 15 Statistical Software; SAS Institute, Inc., Cary, NC, United States). Measures of dispersion (medians, minimum and maximum values) were calculated. Due to the low number of pregnant female, castrated male, and prepubertal male alpacas, differences in hormone medians were compared between mature intact male and non-pregnant female alpacas only. Data were not normally distributed and statistical analysis was performed with non-parametric Kruskal-Wallis test. Data reported as median and range. Significance was set at *p*-value <0.05.

## Results

Hormone concentrations are summarized in Table 1. Similar to other species, such as Mongolian horses (19), cortisol was the predominant steroid (87.7%) in all alpacas. However, unlike Mongolian horses, estradiol was the steroid in lowest concentration (0.1%). Progesterone was the second steroid in abundance (5.1%), which is likely due to the inclusion of pregnant and cycling female alpacas. Testosterone accounted for 3.6% of steroids analyzed, followed by androstenedione (2.5%), and 17-hydroxyprogesterone (1.0%).

**Table 1:** Median (minimum-maximum) hormone concentrations in serum of domestic alpacas.

Physiological status						
Hormone	All (*n*=88)	MI (*n*=30)	MPP (*n*=5)	MC (*n*=2)	FNP (*n*=48)	FP (*n*=3)
Cortisol (μg/dL)	1.0 (<1.0–2.9)	1.0 (<1.0–2.9)	<1.0 (<1.0–1.2)	<1.0 (<1.0)	<1.0 (<1.0–1.5)	<1.0 (<1.0)
Progesterone (ng/ml)	<0.2 (<0.2–4.1)	<0.2 (<0.2–0.4)	<0.2 (<0.2)	<0.2 (<0.2)	<0.2 (<0.2–2.0)	2.2 (1.5–4.1)
Estradiol (pg/ml)	<10.0 (<10.0–137.7)	<10.0 (<10.0–36.8)	<10.0 (<10.0–36.4)	<10.0 (<10.0)	<10.0 (<10.0–137.7)	11.8 (<10.0–17.3)
Testosterone (ng/dL)	<15.0 (<15.0–403.0)	141.5 (22.0–403.0)	19.4 (<15.0–39.2)	<15.0 (<15.0)	<15.0 (<15.0–15.2)	<15.0 (<15.0)
Androstenedione (ng/ml)	0.3 (0.1–2.1)	0.8 (0.2–2.1)	0.4 (0.1–0.5)	0.1 (0.1)	0.1 (0.1–1.0)	0.1 (0.1–0.5)
17-hydroxyprogesterone (ng/ml)	0.1 (<0.1–3.4)	0.2 (<0.1–1.9)	0.1 (<0.1–3.4)	<0.1 (<0.1)	<0.1 (<0.1–1.0)	0.1 (0.1–0.2)
T4 (μg/dL)	6.8 (3.9–12.9)	6.7 (4.3–12.9)	6.7 (4.6–11.8)	7.4 (6.4–8.4)	7.1 (3.9–10.3)	6.1 (4.3–6.8)
T3 (ng/dL)	157.0 (48.7–403.0)	157.0 (48.7–403.0)	126.0 (110.0–181.0)	209.5 (186.0–233.0)	167.0 (77.3–380.0)	168.0 (90.0–179.0)

*n*=number of animals; MI, male intact; MPP male prepubertal; MC, male castrated; FNP, female non-pregnant; FP, female pregnant; < symbol denotes value below assay range.

**Figure 1:**
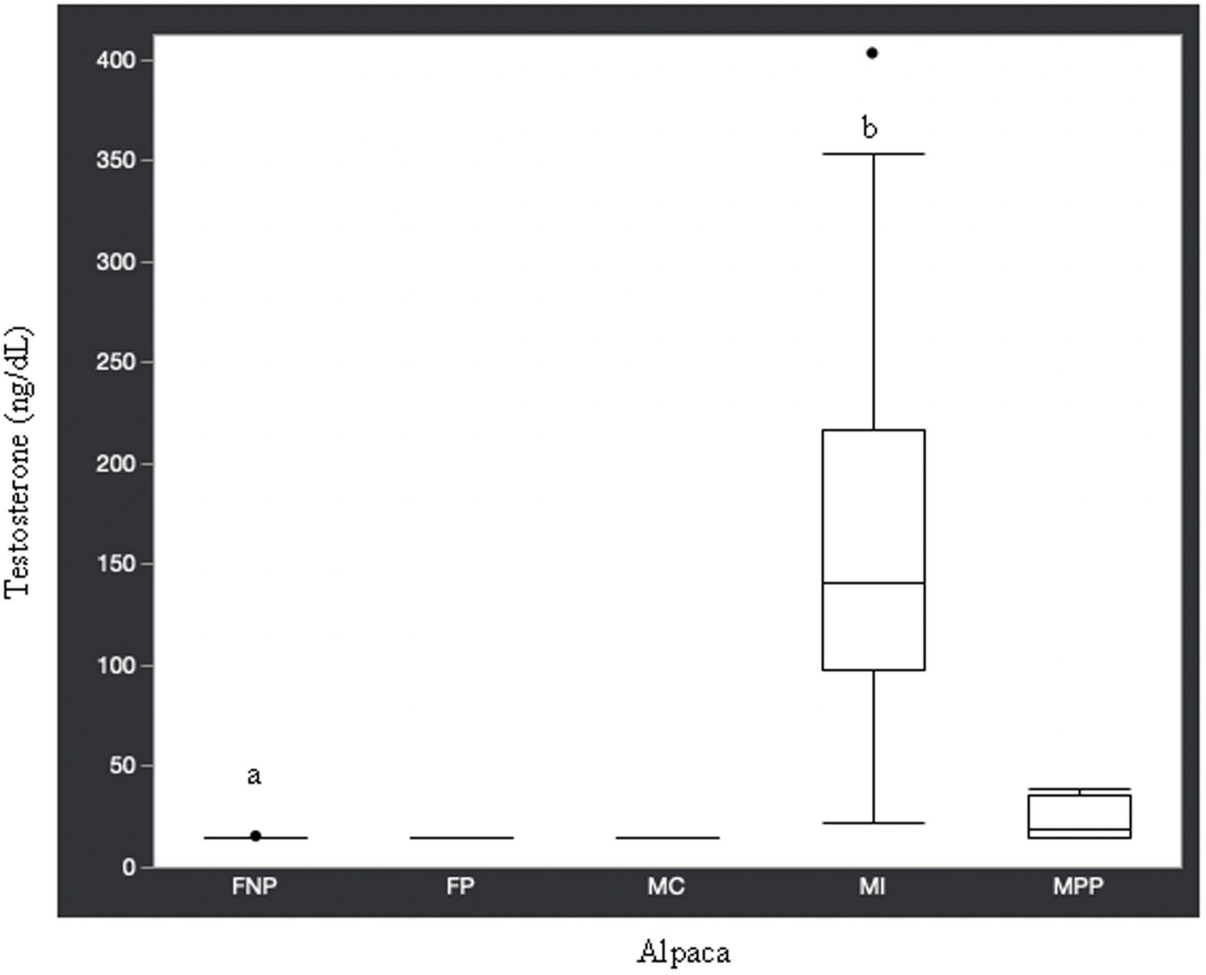
Testosterone concentrations (ng/dL) in serum of domestic healthy alpacas. Different letters (a,b) denote *p* < 0.05 in concentrations between intact male and non-pregnant female alpacas. FNP, female non-pregnant; FP, female pregnant; MC, male castrated; MI, male intact; MPP male prepubertal.

**Figure 2:**
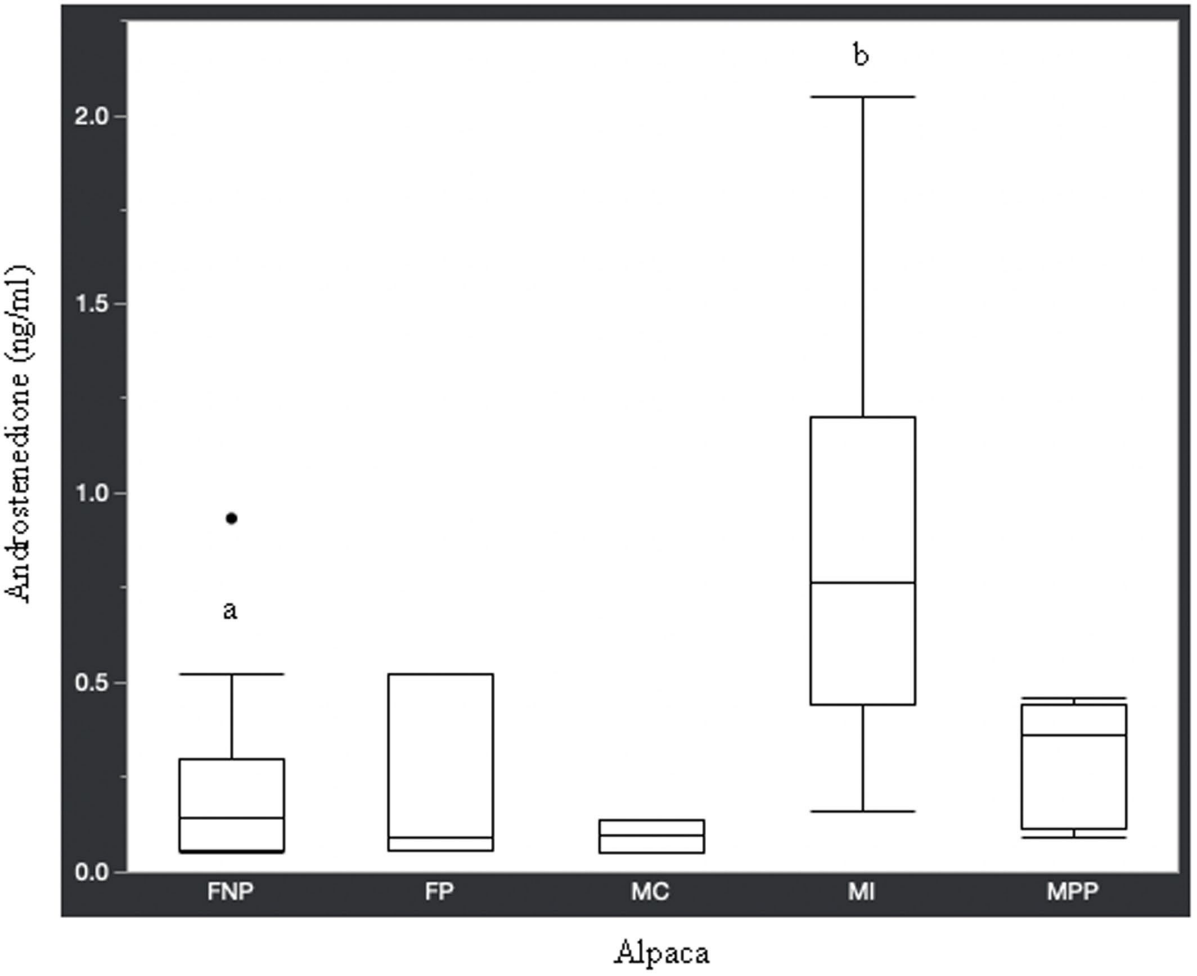
Androstenedione concentrations (ng/ml) in serum of domestic healthy alpacas. Different letters (a,b) denote *p* < 0.05 in concentrations between intact male and non-pregnant female alpacas. FNP, female non-pregnant; FP, female pregnant; MC, male castrated; MI, male intact; MPP male prepubertal.

**Figure 3:**
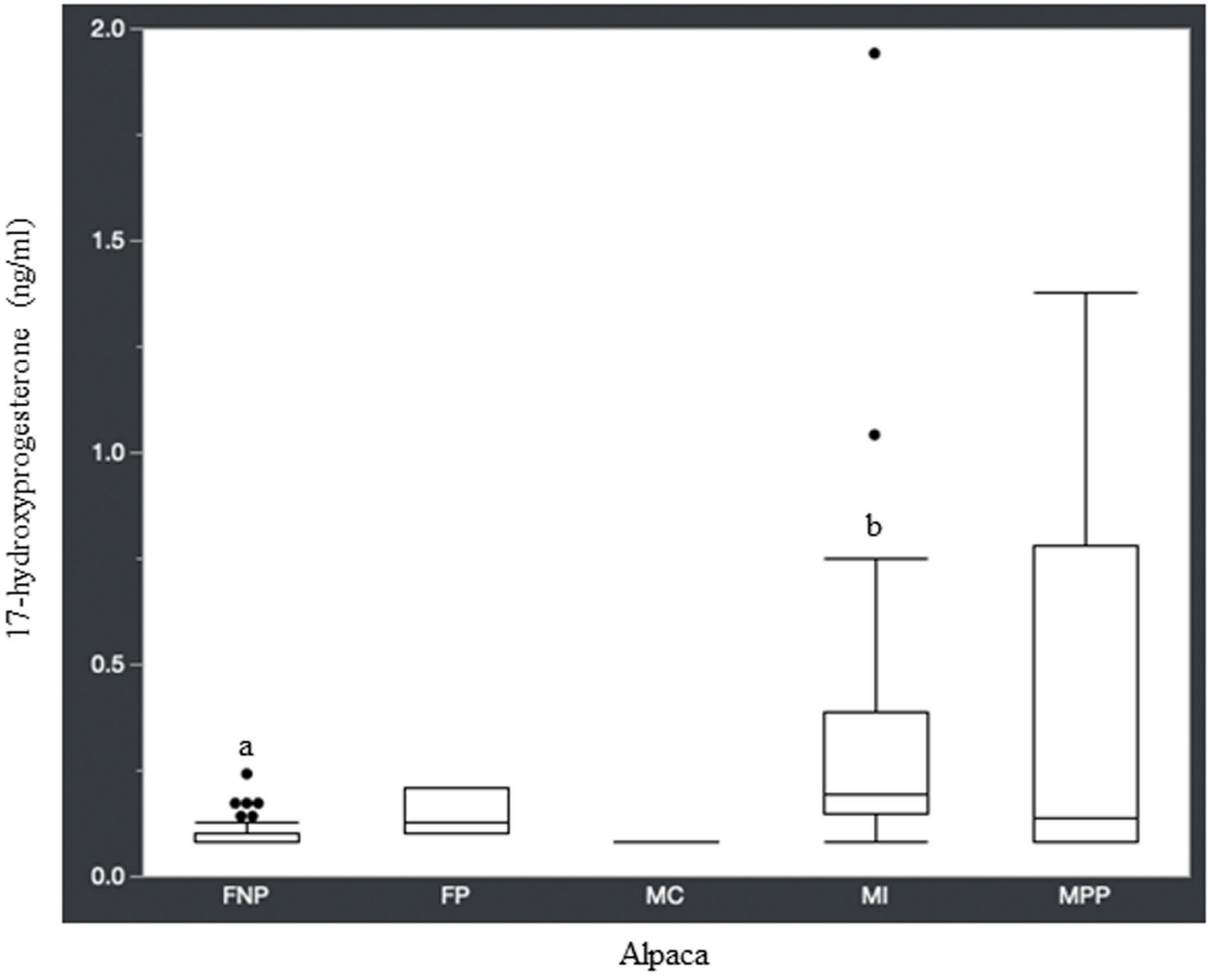
17-hydroxyprogesterone concentrations (ng/ml) in serum of domestic healthy alpacas. Different letters (a,b) denote *p* < 0.05 in concentrations between intact male and non-pregnant female alpacas. FNP, female non-pregnant; FP, female pregnant; MC, male castrated; MI, male intact; MPP male prepubertal.

**Figure 4:**
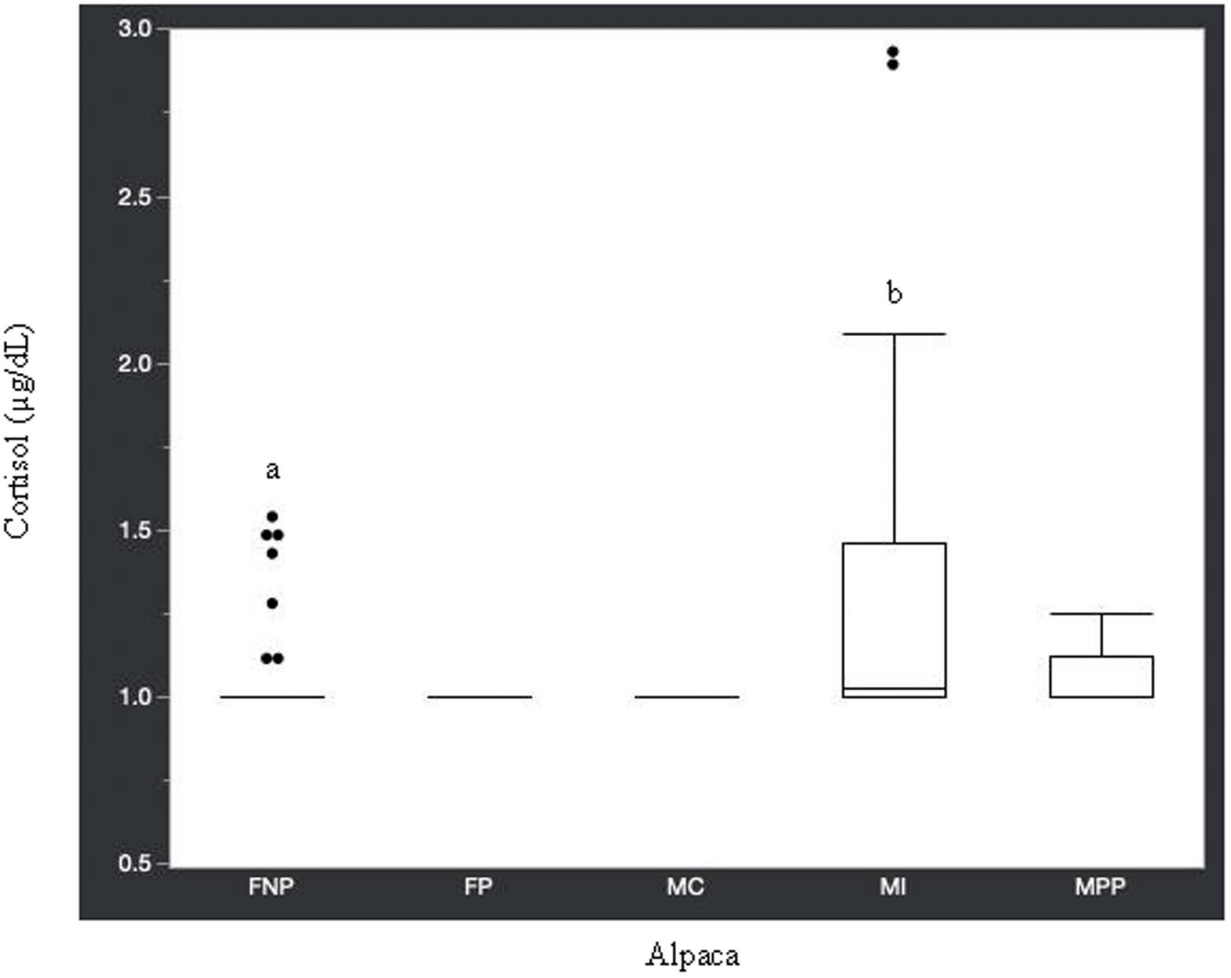
Cortisol concentrations (μg/dL) in serum of domestic healthy alpacas. Different letters (a,b) denote *p* < 0.05 in concentrations between intact male and non-pregnant female alpacas. FNP, female non-pregnant; FP, female pregnant; MC, male castrated; MI, male intact; MPP male prepubertal.

Statistical analysis of the data revealed no significant differences (*p* ≥ 0.05) in median hormone concentrations between intact male and non-pregnant female alpacas, respectively, for progesterone (<0.2 ng/mL and < 0.2 ng/mL), estradiol (<10.0 pg./mL and < 10.0 pg./mL), T4 (6.7 μg/dL and 7.1 μg/dL), and T3 (157.0 ng/dL and 167.0 ng/dL). There were significant differences between mature intact male and non-pregnant female alpacas, respectively, for testosterone (141.5 and < 15.0 ng/dL) (Figure 1), androstenedione (0.8 ng/mL and 0.1 ng/mL) (Figure 2), 17-hydroxyprogesterone (0.2 ng/mL and < 0.1 ng/mL) (Figure 3), and cortisol (1.0 μg/dL and < 1.0 μg/dL) (Figure 4), with males having higher values than females. Testosterone values in the two castrated males were similar to values in female alpacas.

## Discussion

Several articles have been published on alpaca hormone concentrations during pregnancy (7, 8, 20, 21), however, to the authors’ knowledge, this is the first work providing values for several adrenal and gonadal steroids, as well as thyroid hormones, in male and female alpacas. Interestingly, there were no significant differences in median concentrations of progesterone, estradiol, T4, or T3 between intact male and non-pregnant female alpacas. Normal basal progesterone in female camelids has been reported at 0.1 to 0.3 ng/mL (9), which is similar to what we observed in non-pregnant female alpacas as well as intact male alpacas (Table 1). Five of the 48 non-pregnant females had a basal progesterone concentration > 0.20 ng/mL. Since the progesterone level in these females was not associated with pregnancy, it is suspected that their progesterone values reflected luteal activity. The alpaca is an induced ovulator, and as such, ovulation is normally initiated by breeding to an intact or vasectomized male (7). It is not known if any of these farms housed vasectomized male alpacas which may have caused these females to ovulate however, one non-pregnant female in this study with a progesterone value of 0.72 ng/mL was bred to an intact male but aborted the fetus.

Similar to progesterone, the median estradiol concentrations were similar between male and female alpacas. In addition to alpacas, male dogs and female dogs in anestrus have been reported to have similar estradiol values (17). Normal basal estradiol concentration reported in female camelids is <10.0 pg./mL, with an increase in concentration with the development of a mature ovarian follicle (9). In alpacas, ovarian follicles grow in waves, which tend to overlap (7). Therefore, a dominant follicle in one wave may be regressing as a follicle from another wave becomes dominant (7). The minimum value of estradiol in all alpacas in our study was <10.0 pg./mL, while higher values in some of the non-pregnant females (Table 1) were likely due to ovarian follicle maturation.

Thyroid hormone values in alpacas have been reported by Anderson and Silveira (22). They found no significant differences in mean T4 and T3 concentrations between male and female alpacas ≥2 years of age, which is in agreement with median results from our study (Table 1). Hyperplasia (23) and carcinoma (24) of the thyroid gland have been described in a llama however, reports in the literature on thyroid disease in alpacas is scarce. Interestingly, alpaca T4 values in this study are approximately 4-fold higher than some other species such as equine, canine, and feline, while T3 is approximately 3-to 6-fold higher than the aforementioned species (unpublished data from our laboratory). The reason for higher thyroid values in alpaca compared to other species is not known. Alpacas in this study had free access to commercial mineral products however, it is not known how these formulations compare with those provided to other species. Fowler and Zinkl (25) reported T4 values in llamas that were up to 10-times higher than T4 in cattle and horses. Mean T4 values in other camelids, such as camels (26) and llamas (10), are higher than median values determined in our study however these mean camel and llama values fall within the minimum to maximum T4 values for all alpacas in the current study.

Contrary to the aforementioned hormones, there were significant differences in testosterone, androstenedione, 17-hydroxyprogesterone, and cortisol concentrations between intact male and intact non-pregnant female alpacas. The higher concentration of androgens in intact males versus females is expected since testicles are the main source of testosterone and androstenedione. Though testicles are the main source of testosterone, there is an article (27) in which testosterone was elevated (969.1 pg./mL or 96.9 ng/dL) in an intact female alpaca with an ovarian interstitial cell tumor. The authors of that article determined testosterone values (11.7–62.1 pg./mL or 1.2–6.2 ng/dL) in healthy adult intact female alpacas for comparison to the affected animal using radioimmunoassay methodology. The maximum value we observed in intact female alpacas was 15.0 ng/dL, which is approximately double the maximum determined by Gilbert et al. (27). Differences in reference values are not unexpected since it is known that they may differ from laboratory to laboratory depending on the methodology used for analysis.

Along with testosterone and androstenedione, 17-hydroxyprogesterone was higher in male alpacas (Table 1). Interestingly, a study in humans found that plasma concentration of 17-hydroxyprogesterone was significantly higher in healthy men than in women in the follicular phase of the menstrual cycle, and that the testicular Leydig cell produced 90% of 17-hydroxyprogesterone in these men (28). It is known that 17-hydroxyprogesterone is a precursor for androstenedione, with conversion via the 17,20-lyase enzyme. Androstenedione, in turn, can be converted to testosterone via the 17β-hydroxysteroid dehydrogenase enzyme, which may help explain the higher concentration of 17-hydroxyprogesterone in mature intact male alpacas compared to non-pregnant female alpacas.

Approximately 74% of the alpacas in this current study had cortisol concentrations <1.0 μg/dL, which is similar to the mean basal value (0.75 μg/dL) reported in a study by Anderson et al. (29). In the same study (29), cortisol increased to a mean of 1.63 μg/dL in male and 1.95 μg/dL in females after 30 min of transportation then returned to basal level 4 h later. It is possible that the higher cortisol concentrations in some of the animals in our study were related to stress caused by restraint for blood collection. However, Anderson et al. (29) concluded that physical restraint did not cause psychological stress in their study alpacas since hypercortisolemia was not found in the blood sample collected 4 h post-transportation. Therefore, it is reasonable to assume the higher cortisol values in some of the male and female alpacas in the current study are due to individual variation and not stress related.

A limitation to this study is the Immulite 1,000 instrument, used for several of the hormone analyses, is no longer available in the United States, however assay kits are still commercially available. Analyses were conducted 6 years later on the Immulite 2000 XPi instrument, however, several samples did not have sufficient serum for re-analysis. Comparative data is available (Supplementary material).

In conclusion, the hormone values determined in this current study add to the limited information available in the literature on alpacas. Knowledge of hormone concentrations in adult male and female alpacas in the Southeastern United States will aid veterinarians in the diagnosis of endocrinopathies in this species of camelid, and further elucidate physiological characteristics of camelid family members.

## Data availability statement

The raw data supporting the conclusions of this article will be made available by the authors, without undue reservation.

## Ethics statement

The animal studies were approved by Institutional Animal Care and Use Committee, University of Tennessee. The studies were conducted in accordance with the local legislation and institutional requirements. Written informed consent was obtained from the owners for the participation of their animals in this study.

## Author contributions

KF: Conceptualization, Validation, Writing – original draft, Writing – review & editing. LG: Writing – review & editing. HE: Writing – review & editing. AE-V: Formal analysis, Writing – review & editing. AL: Resources, Writing – review & editing. RV: Resources, Writing – review & editing.

## References

[1] Fowler ME. Husbandry and diseases of camelids. Rev Sci Tech Off Int Epiz. (1996) 15:155–69. doi: 10.20506/rst.15.1.912 8924702

[2] D’Alterio GL Knowles TG Eknaes EI Loevland IE Foster AP. Postal survey of the population of south American camelids in the United Kingdom in 2000/01. Vet Rec. (2006) 158:86–90. doi: 10.1136/vr.158.3.86 16428662

[3] Gillespie RAM Williamson LH Terrill TH Kaplan RM. Efficacy of anthelmintics on south American camelid (llama and alpaca) farms in Georgia. Vet Parasitol. (2010) 172:168–71. doi: 10.1016/j.vetpar.2010.04.009, PMID: 20462700

[4] Franceschi V Jacca S Sassu EL Stellari FF van Santen VL Donofrio G. Generation and characterization of the first immortalized alpaca cell line suitable for diagnostic and immunization studies. PLoS One. (2014) 9:e105643. doi: 10.1371/journal.pone.0105643, PMID: 25140515 PMC4139384

[5] Gegner L Sharp H. Llamas and alpacas on the farm National Sustainable Agriculture Information Service: National Center for Appropriate Technology (2012). Available at: https://www.attra.ncat.org

[6] United States Department of Agriculture. (2017) Census of Agriculture. Available at: https://www.nass.usda.gov/agcensus (Accessed December 8, 2020).

[7] Bravo W. Reproductive endocrinology of llamas and alpacas. Vet Clin North Am Food Anim Pract. (1994) 10:265–79. doi: 10.1016/S0749-0720(15)30561-2 7953960

[8] Raggi LA Ferrando G Parraquez VH MacNiven V Urquieta B. Plasma progesterone in alpaca (*Lama pacos*) during pregnancy, parturition and early postpartum. Anim Reprod Sci. (1999) 54:245–9. doi: 10.1016/S0378-4320(98)00156-0 10090567

[9] Cotton T. Reproductive hormones in camelids. Proc. of 25th NAVC conference. Orlando, Florida, 19–23(2008) 290–293.

[10] Smith BB Pearson EG Leon J. Evaluation of normal triiodothyronine and tetraiodothyronine concentrations in llamas (*Lama glama*). Am J Vet Res. (1989) 50:1215–9. PMID: 2782704

[11] Arias N Requena M Palme R. Measuring faecal glucocorticoid metabolites as a non-invasive tool for monitoring adrenocortical activity in south American camelids. Anim Welf. (2013) 22:25–31. doi: 10.7120/09627286.22.1.025

[12] Lennox AM Chitty J. Adrenal neoplasia and hyperplasia as a cause of hypertestosteronism in two rabbits. J Exotic Pet Med. (2006) 15:56–8. doi: 10.1053/j.jepm.2005.11.009

[13] Chiaramonte D Greco DS. Feline adrenal disorders. Clin Tech Small Anim Pract. (2007) 22:26–31. doi: 10.1053/j.ctsap.2007.02.004 17542194

[14] Camps T Amat M Manteca X. A review of medical conditions and behavioral problems in dogs and cats. Animals. (2019) 9:1133. doi: 10.3390/ani9121133, PMID: 31842492 PMC6941081

[15] Cecere J Purswell B Panciera D. Levothyroxine supplementation in hypothyroid bitches during pregnancy. Theriogenology. (2020) 142:48–53. doi: 10.1016/j.theriogenology.2019.09.036, PMID: 31574400

[16] Rosenthal KL Peterson ME. Evaluation of plasma androgen and estrogen concentrations in ferrets with hyperadrenocorticism. J Am Vet Med Assoc. (1996) 209:1097–102. PMID: 8800255

[17] Frank LA Rohrbach BW Bailey EM West JR Oliver JW. Steroid hormone concentration profiles in healthy intact and neutered dogs before and after cosyntropin administration. Domest Anim Endocrinol. (2003) 24:43–57. doi: 10.1016/S0739-7240(02)00204-7, PMID: 12450624

[18] Fecteau KA Deeb BJ Rickel JM Kelch WJ Oliver JW. Diagnostic endocrinology: blood steroid concentrations in neutered male and female rabbits. J Exotic Pet Med. (2007) 16:256–9. doi: 10.1053/j.jepm.2007.09.003

[19] Haffner JC Fecteau KA Eiler H Tserendorj T Hoffman RM Oliver JW. Blood steroid concentrations in domestic Mongolian horses. J Vet Diagn Investig. (2010) 22:537–43. doi: 10.1177/104063871002200407, PMID: 20622223

[20] Knight TW Ridland M Scott I Death AF Wyeth TK. Foetal mortality at different stages of gestation in alpacas (*Lama pacos*) and the associated changes in progesterone concentrations. Anim Reprod Sci. (1995) 40:89–97. doi: 10.1016/0378-4320(95)01415-V

[21] Aba MA Bravo PW Forsberg M Kindahl H. Endocrine changes during early pregnancy in the alpaca. Anim Reprod Sci. (1997) 47:273–9. doi: 10.1016/S0378-4320(97)00028-6, PMID: 9360766

[22] Anderson DE Silveira F. Effect of age and gender on serum concentration of triiodothyronine and tetraiodothyronine (thyroxine) in alpacas (lama pacos). J Anim Vet Adv. (2003) 2:626–9.

[23] Hamir AN Timm KI. Nodular hyperplasia and cysts in thyroid glands of llamas (*Lama glama*) from north-West USA. Vet Rec. (2003) 152:507–8. doi: 10.1136/vr.152.16.507, PMID: 12733562

[24] Carrasco RA Verhoef J Leonardi CEP Lanigan EE Adams GP. Bilateral thyroid follicular compact-cellular carcinoma in a llama. J Vet Diagn Investig. (2019) 31:913–6. doi: 10.1177/1040638719882734, PMID: 31646945 PMC6900731

[25] Fowler ME Zinkl JG. Reference ranges for hematologic and serum biochemical values in llamas (*Lama glama*). Am J Vet Res. (1989) 50:2049–53. PMID: 2610431

[26] Saeb M Baghshani H Nazifi S Saeb S. Physiological response of dromedary camels to road transportation in relation to circulating levels of cortisol, thyroid hormones and some serum biochemical parameters. Trop Anim Health Prod. (2010) 42:55–63. doi: 10.1007/s11250-009-9385-9, PMID: 19544085

[27] Gilbert R Kutzler M Valentine BA Semevolos S. Hyperandrogenism from an ovarian interstitial-cell tumor in an alpaca. J Vet Diagn Investig. (2006) 18:605–7. doi: 10.1177/104063870601800616, PMID: 17121093

[28] Strott CA Yoshimi T Lipsett MB. Plasma progesterone and 17-hydroxyprogesterone in normal men and children with congenital adrenal hyperplasia. J Clin Invest. (1969) 48:930–9. doi: 10.1172/JCI106052, PMID: 4305376 PMC322302

[29] Anderson DE Grubb T Silveira F. The effect of short duration transportation on serum cortisol response in alpacas (llama pacos). Vet J. (1999) 157:189–91. doi: 10.1053/tvjl.1998.0270, PMID: 10204416

